# A Novel Insight on Endotyping Heterogeneous Severe Asthma Based on Endoplasmic Reticulum Stress: Beyond the “Type 2/Non-Type 2 Dichotomy”

**DOI:** 10.3390/ijms20030713

**Published:** 2019-02-07

**Authors:** Jae Seok Jeong, So Ri Kim, Seong Ho Cho, Yong Chul Lee

**Affiliations:** 1 Department of Internal Medicine, Research Center for Pulmonary Disorders, Chonbuk National University Medical School, Jeonju 54907, Korea; jeongjs@jbnu.ac.kr (J.S.J.); sori@jbnu.ac.kr (S.R.K.); 2 Research Institute of Clinical Medicine of Chonbuk National University–Biomedical Research Institute of Chonbuk National University Hospital, Jeonju 54907, Korea; 3 Division of Allergy and Immunology, Internal Medicine, Morsani College of Medicine, University of South Florida, Tampa, FL 33618, USA; schonwubf@gmail.com

**Keywords:** severe asthma, heterogeneity, endotype, endoplasmic reticulum stress

## Abstract

Severe asthma is an extremely heterogeneous clinical syndrome in which diverse cellular and molecular pathobiologic mechanisms exist, namely endotypes. The current system for endotyping severe asthma is largely based on inflammatory cellular profiles and related pathways, namely the dichotomy of type 2 response (resulting in eosinophilic inflammation) and non-type 2 response (reinforcing non-eosinophilic inflammation involving neutrophils or less inflammatory cells), forming the basis of a development strategy for novel therapies. Although specific subgroups of type 2 severe asthma patients may derive benefit from modern precision medicine targeting type 2 cytokines, there is no approved and effective therapeutic agent for non-type 2 severe asthma, which comprises nearly 50% of all asthma patients. Importantly, the critical implication of endoplasmic reticulum (ER) stress and unfolded protein response—in close relation with several pivotal cellular immune/inflammatory platforms including mitochondria, NLRP3 inflammasome, and phosphoinositide 3-kinase-δ—in the generation of corticosteroid resistance is now being increasingly demonstrated in numerous experimental settings of severe asthma. Consistent with these findings, recent clinical data from a large European severe asthma cohort, in which molecular phenotyping as well as diverse clinical and physiological parameters from severe asthmatic patients were incorporated, suggest a brand new framework for endotyping severe asthma in relation to ER-associated mitochondria and inflammasome pathways. These findings highlight the view that ER stress-associated molecular pathways may serve as a unique endotype of severe asthma, and thus present a novel insight into the current knowledge and future development of treatment to overcome corticosteroid resistance in heterogeneous severe asthma.

## 1. Introduction

**Figure 1:**
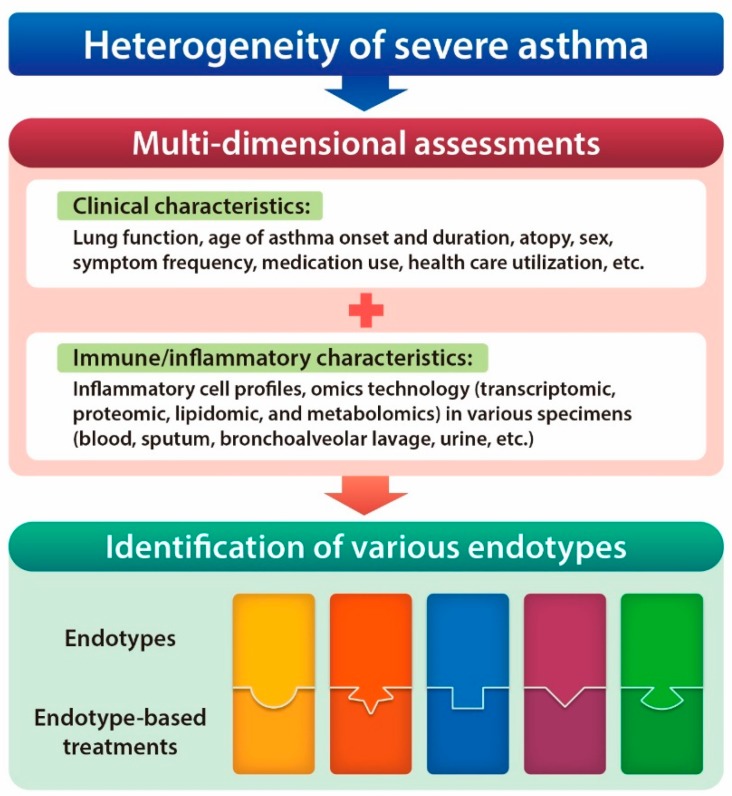
Multi-dimensional approaches involving both clinical characteristics and immune/inflammatory profiles are required for proper identification of diverse endotypes and subsequent development of endotype-based treatments in heterogeneous severe asthma.

Since the early investigational approaches to improve our understanding of bronchial asthma in the late 1990s, many researchers have focused on the heterogeneity of the disease, which may cause different treatment responses to any pharmacologic intervention. Since then, through numerous insightful basic and clinical studies, it is now becoming evident that chronic inflammation of the airways in bronchial asthma can be driven by different pathobiologic mechanisms (i.e., endotypes) possessing unique cellular and molecular inflammatory profiles [1,2]. This concept of disease is particularly important when investigating severe asthma, because numerous severe asthma cohorts in the United States and Europe [3] have consistently shown its heterogeneity in the context of diverse clinical; physiologic; or, more recently, inflammatory characteristics. Moreover, defining those disease-driving mechanisms may provide insights for targeted and personalized treatment in each population of severe asthmatics [2], given that severe asthma represents the majority of asthma morbidity and healthcare costs [4]. For instance, early clustering analysis using clinical data from the Severe Asthma Research Program (SARP) cohort revealed five distinct clinical phenotypes of severe asthma patients who differ in lung function, age of onset and duration, atopy status, sex, symptom frequency, medication use, and healthcare utilization [5]. However, later assignment of sputum inflammatory profiles based on sputum granulocytes [6] to subjects within those clusters showed the lack of association between the inflammatory cell profiles and clinical clusters [7]. These findings emphasize that correct subtyping of severe asthma and subsequent development of novel therapeutic agents should consider information on the underlying pathobiology, as well as diverse clinical parameters (Figure 1).

However, whether the inflammatory cell profile reflects the underlying pathobiological processes needs to be further verified. A great number of distinct cell types contribute to the immunopathobiology of bronchial asthma [8]. Each cell type may play a unique role in a particular stage of the disease process, and they are tightly intertwined with the others during the whole pathogenesis. Nonetheless, inflammatory cellular profiles of asthma, particularly the severe form, now principally fall into being eosinophilic and non-eosinophilic in nature in many clinical studies or real medical practice, partly owing to their predominance among various specimens [9]. In addition, those granulocytic cell types are also relatively easy to recognize without further immunophenotyping of cells. This led to the current dichotomy of type 2 response (resulting in eosinophilic inflammation) and non-type 2 response (reinforcing non-eosinophilic inflammation involving neutrophils or less inflammatory cells, namely the pauci-granulocytic response) in severe asthma pathogenesis. On the basis of this dichotomy, biologic therapies interfering with type 2-related or non-type 2 inflammatory pathways are clinically available, and some are actively under development [2]. Notably, in a recent clustering analysis of SARP cohort, most severe forms of asthma have been reported to possess the mixed inflammatory cell nature, comprising both eosinophils and neutrophils [10]. In addition, the heterogeneity of the underlying biological pathways is prominent in the mixed granulocytic phenotype of asthma [11], implying that inflammatory cell profiles may not correctly reflect the underlying pathobiology, particularly in many severe asthma patients. Consequently, there is an urgent need for more advanced endotyping approaches that incorporate universal biological mechanisms of various cells. 

In the context of cell biology, subcellular organelles are distributed throughout the eukaryotic cells and each organelle has a specific function to maintain cellular homeostasis. Among them, the endoplasmic reticulum (ER) and mitochondria have increasingly drawn attention with regard to their broad involvement of cellular immune/inflammatory responses under physiologic and pathologic conditions. In particular, their contributions to the pathogenesis of corticosteroid (CS)-resistant severe asthma have recently been appreciated through numerous basic studies on this issue [12,13]. Importantly, increasing data from large and well-designed human clinical cohorts of severe asthma also support this concept of disease pathogenesis [11]. In this review, we will summarize the recent advancement of our understanding in the pathobiology of severe asthma, focusing particularly on the ER, mitochondria, and the related cellular platforms of inflammation, thereby presenting a new framework for the development of specific treatments for severe asthma.

## 2. Type 2 and Non-Type 2 Immune Responses in the Heterogeneity of Severe Asthma

Previous studies have revealed that the diverse inflammatory pathways implicated in severe asthma may fall into type 2 or non-type 2 inflammation, according to the underlying immune/inflammatory mechanisms. However, this simplified categorization still does not seem to correctly reflect the underlying pathobiologic processes of CS-resistant inflammation in severe asthma, which are more complex than we expected and may involve both type 2 and non-type 2 pathways simultaneously to a variable extent during the chronic course of the disease process. 

## 3. Type 2 Inflammation: Allergic and Non-Allergic Eosinophilic Airway Inflammation

Classically, bronchial asthma has been regarded as a type 2 helper T cell (TH2 cell)-mediated disorder of the lungs, which coincides with the presence of eosinophilic inflammation (i.e., observable eosinophilic airway inflammation in bronchoalveolar lavage (BAL) fluid, induced sputum, or bronchial biopsy samples). In this concept of bronchial asthma pathogenesis, the presence of serum allergen-specific immunoglobulin E (IgE) related to atopy/allergy is the hallmark of the adaptive TH2 response, and increased numbers of type 2 cytokine (i.e., interleukin 4 (IL-4), IL5, and IL-13)-producing CD4 positive T cells, which are stimulated by dendritic cells, contribute to eosinophilic airway inflammation and airway hyper-responsiveness (AHR). This immune pathway is known to be CS-sensitive and an essential mechanism that underlies many allergic diseases such as allergic asthma, allergic rhinitis, and atopic dermatitis [8,14]. Experimentally, this type of asthma can be successfully reproduced by inhalation of ovalbumin (OVA), a prototypical mouse allergen, after extrapulmonary sensitization to OVA with alum (as immunologic adjuvant). In this murine model of asthma, IL-4 is essential in class switching of immunoglobulins produced by plasma cells and the subsequent development of adaptive TH2 and humoral immunity (production of IgE antibodies to allergens). IL-13 is thought to be important in the maintenance of AHR and mucin production [15]. Importantly, the eosinophilia in lung tissues is mainly driven by IL-5 and is thought to be implicated in the induction of the adaptive T cell response [16,17], AHR [18], and airway remodeling [19]. Currently, several biomarkers of TH2-mediated type 2 inflammation have been demonstrated including blood eosinophilia, fractional exhaled nitric oxide [20], and blood levels of IL-25 [21] and blood periostin [22], all of which have been shown to correlate well with airway eosinophilia. More recently, researchers have revealed that pulmonary type 2 inflammation with eosinophilia can also be a result of acute or chronic activation of type 2 innate lymphoid cells (ILC2), which is induced by IL-25, IL-33, and thymic stromal lymphopoietin (TSLP) produced mainly by airway epithelial cells (epithelium-derived cytokines) in a T cell-independent manner. Experimentally, ILC2 can be activated readily after allergen exposure by a single exposure to proteolytic allergens (e.g., *Alternaria* species) [23] and can also be stimulated chronically by epithelial activation (through direct injury or activation of pattern-recognition receptors) and subsequent production of epithelium-derived cytokines in association with environmental exposure to pollutants, irritants, fungi, and viruses, thereby producing IL-5 and IL-13, causing lung eosinophilia and AHR regardless of atopy/allergy [8]. ILC2 expresses the same chemokine receptors including chemokine receptors expressed on TH2 cells [24], CRTH2 (prostaglandin D2 receptor), and cysteinyl leukotriene receptor 1 [23], enabling this cell type to be an active participant during the entire pulmonary type 2 inflammation process. Furthermore, in contrast to TH2 cell-mediated inflammation, the ILC2-related type 2 pathway is increasingly known to be CS-resistant in nature, suggesting that ILC2-mediated type 2 inflammation may be implicated in severe asthma and acute exacerbation of asthma [25,26]. However, at the same time, ILC2 may also facilitate the polarization of naïve CD4-positive T cells to TH2 cells partly through releasing cytokines such as IL-13 [27] and possibly acting as antigen-presenting cells [28]. Taken together, the aforementioned cellular diversity contributing to pulmonary type 2 inflammation may explain why the blockade of type 2 cytokines is efficacious in non-allergic type 2 inflammation severe asthma with increased levels of blood eosinophils [29,30,31]. Furthermore, differences in the extent of the relative contribution between TH2 cells and ILC2 cells render pulmonary type 2 inflammation more complex with regard to treatment response and clinical outcomes, leading to clinical heterogeneity within type 2 eosinophilic severe asthma.

## 4. Non-Type 2 Inflammation: Neutrophilic Airway Inflammation in Association with Type 2 Immune Response

Since initial studies demonstrating that a considerable proportion of bronchial asthma may be driven by alternative forms of airway inflammation other than TH2-mediated inflammation [32,33], researchers have found that asthma patients with non-type 2 inflammation generally manifest adult-onset and less CS-responsive disease, have lower lung function clinically, and frequently possess neutrophilic airway inflammation [34,35]. The overall proportion of this subgroup of asthma patients is estimated to be approximately 50% of all asthma patients, given that the blockade of type 2 cytokine did not show beneficial effects in non-phenotyped and overall groups of patients who probably comprise both type 2 and non-type 2 asthma [36]. Subsequent studies have revealed that neutrophilic inflammation in non-type 2 asthma may result from the activation of both TH1 (type 1) and TH17 (type 17) cytokines [37,38,39], although this is not fully understood. Experimentally, adoptive transfer of OVA-specific TH17 cells to mice resulted in neutrophil influx to the lungs through the action of a neutrophil chemoattractant IL-8, which was not ameliorated by treatment with dexamethasone [38]. Moreover, expression of TH17-related cytokines including IL-17A and IL-17F has been demonstrated to be correlated with asthma severity in human airway tissue [37]. TH1/IFN-γ also seems to be crucially implicated in TH17-associated neutrophilic inflammation of CS-resistant severe asthma. Patients with severe asthma possess more IFN-γ-positive and IL-17A-positive CD4-positive T cells in BAL cells [40] and increased production of both IL-17A and IFN-γ by CD8-depleted PBMCs from patients with CS-resistant asthma compared with patients with CS-sensitive asthma [41]. Interestingly, one recent study demonstrated that the numbers of TH1-enriched CD4-positive T cells in BAL cells was inversely correlated with the percent predicted forced expiratory volume in 1 s (FEV1) [42], indicating the unique role of TH1 inflammation in severe asthma. In fact, simultaneous activation of type 1/type 17 inflammation has been reported in a clustering analysis using sputum transcriptomics in the Unbiased Biomarkers for the Prediction of Respiratory Disease Outcomes (U-BIOPRED) cohort of severe asthma [11]. Furthermore, according to the recent clustering analysis involving 112 clinical, physiologic, and inflammatory variables in the SARP cohort, combined eosinophilic/neutrophilic inflammation may be a biomarker of the most severe form of asthma [10]. Considering that both TH1 and TH17 can also promote type 2 inflammation experimentally [43,44,45,46], these findings are consistent with the hypothesis that intricate interaction between type 1 and type 17 immune response in a background of variable extent of type 2 immunity underlies the heterogeneous inflammatory nature of CS-resistant severe asthma [47,48]. In this context, it is predictable that a therapeutic strategy targeting a single mediator of non-type 2 immune response such as IL-17A (brodalumab, a human anti-IL-17RA monoclonal antibody) does not produce a remarkable treatment effect in subjects with moderate to severe asthma in clinical studies [49].

### 4.1. A New Perspective on Endotyping Heterogeneous Severe Asthma: Implication of Subcellular Organelles

As described above, there may be further complex interactions between various cell types within each inflammatory endotype, leading to the vast clinical heterogeneity of severe asthma. Thus, this dichotomy can be less useful in endotyping and subsequent development of endotype-driven therapy for severe asthma. In fact, therapeutic tools targeting a specific mediator or single immune/inflammatory pathway would lack broad clinical efficacy, although they might be effective for a certain phenotype of severe asthma patients, which partly explains why the cure for severe asthma is still challenging. Recently, the body of evidence has highlighted the role of functional disturbances in subcellular organelles in generating a myriad of immune and inflammatory processes of severe allergic inflammation, which involves broad cell types in pulmonary immunology [13,50]. Importantly, the restoration of their functionality is likely to be an ideal target in the development of a therapeutic agent in severe asthma, because it is physiological and thus there might be less serious adverse effects, rather than blocking or eliminating targets. Furthermore, the functionality of subcellular organelles is closely associated with each other and with several critical immune/inflammatory platforms known to be key inducers of CS-resistant allergic lung inflammation of severe asthma. In this article, we will focus on the interrelationship between these organelles, but not cover in detail the various canonical and non-canonical aspects of ER stress and unfolded protein response (UPR), which have been extensively reviewed elsewhere [12,13].

### 4.2. ER Stress as a Potential Endotype of Severe Asthma

As a cellular protein folding factory, the ER is highly sensitive to diverse stresses that interfere with cellular energy levels, Ca^2+^ concentration, and cellular redox state, thus perturbation of which causes imbalance in protein homeostasis frequently occurs under various pathologic conditions [51]. Moreover, in the pathogenesis of chronic inflammatory diseases such as bronchial asthma, diverse cell types produce large amounts of secretory and membrane proteins to communicate with other cell types not only for their own defense, but also for the generation of an efficient and integrated immune/inflammatory response, which essentially relies on the proper function of the ER [52]. Therefore, it is no surprise that the ER intersects on multiple levels with immune and inflammatory responses, thereby playing an important role as a critical sensor of cellular stresses, as well as a regulator of the inflammatory process. As for the respiratory system, numerous resident structural cells (e.g., epithelial cells and tissue-resident dendritic cells) and inflammatory cells (e.g., granulocytes such as eosinophils and macrophages) depend on proper ER function in regard to various aspects of normal physiology (e.g., cellular differentiation and secretion of immunomodulatory mediators) [13,53]. Moreover, at the same time, from a pathologic viewpoint, many environmental triggers of asthma including air pollutants, cigarette smoke, allergens, and bacteria and viruses are also known to induce ER stress and UPR in the lung [52], thereby being implicated in the initiation of pathologic immune/inflammatory processes. 

In particular, a growing body of evidence indicates that ER stress and UPR are closely associated with CS-resistant allergic lung inflammation, apart from predominant inflammatory cell phenotypes, and possess potential as a novel endotype for severe asthma. We previously demonstrated that neutrophilic allergic lung inflammation and associated ER stress in the lungs of mice, induced by OVA/LPS sensitization followed by OVA challenge (OVALPS-OVA model), were remarkably attenuated by treatment with a potent ER stress regulator, 4-phenylbutyric acid (4-PBA) [54]. However, dexamethasone treatment failed to improve neutrophil-dominant allergic lung inflammation as well as ER stress in OVALPS-OVA mice. Interestingly, there were increased mixed type 1/type 17 immune responses in a background of type 2 inflammation (i.e., increases in IFN-γ/IL-17/type 2 cytokines including IL-4, IL-5, and IL-13) of the lungs from OVALPS-OVA mice, implying that this murine model may represent a typical endotype of non-type 2 severe asthma. Consistent with these results, induction of ER stress using a well-known ER stress inducer, tunicamycin, aggravates ER stress and increases the expression of pro-inflammatory mediators associated with neutrophilic inflammation (e.g., IL-6, IL-8, and TNF-α) through PERK–ATF4–CHOP signaling in mouse bronchial epithelial cells and lung tissue of a neutrophilic severe asthma model [55]. In addition, ER stress may also be a critical player in type 2 severe asthma. A recent study has shown that pulmonary ER stress and UPR-related markers are significantly elevated in a fungus (*Aspergillus fumigatus*, Af)-induced CS-resistant asthma murine model [56]. At the same time, Af-exposed mice display typical type 2 asthma including eosinophil-predominant allergic lung inflammation and an increase in the levels of serum total/Af-specific IgE and pulmonary type 2 cytokines (IL-4, IL-5, and IL-13). Notably, the administration of 4-PBA remarkably improves severe type 2 asthmatic features as well as ER stress in Af-exposed mice, while dexamethasone fails to improve these, suggesting that ER stress also influences eosinophilic type 2 severe asthma. This is further verified by the finding that GRP78, a representative ER stress marker, is significantly increased in lung tissue from patients with allergic bronchopulmonary aspergillosis (ABPA), which is a severe spectrum of type 2 allergic responses against fungi [12,57]. Taken together, ER stress may be critically implicated in the pathogenesis of CS-resistant severe allergic lung inflammation, irrespective of the underlying inflammatory cellular phenotype. This finding highlights the potential of ER stress as a novel endotype of severe asthma.

Indeed, studies on the mechanism through which ER stress can be linked to CS-resistance in the lung have unveiled several molecular networks. Among them, ER stress-associated nuclear factor (NF)-κB activation, a master regulator of inflammation, may be important [58]. In both OVALPS-OVA mice and Af-exposed mice [54,56], there is increased nuclear translocation of NF-κB p65 in the lungs, and inhibition of ER stress results in a decrease in OVALPS- and Af-induced NF-κB nuclear translocation. In particular, administration of NF-κB inhibitor, BAY 11-7085, markedly attenuates CS-resistant severe asthma features [56], emphasizing its role in mediating CS-resistance. In view of the mechanisms that may link ER stress-associated NF-κB activation and CS resistance, NF-κB is likely to be associated with double-stranded RNA (dsRNA)-activated serine/threonine kinase R (PKR), an essential component of the innate antiviral response and, in relation to ER stress, PKR phosphorylates a component of UPR, eukaryotic initiation translation factor 2α (eIF2α). Our preliminary data showed that the administration of poly I:C, a synthetic analog of dsRNA, aggravated all severe asthmatic features of the CS-resistant neutrophilic OVALPS-OVA model, resembling CS-resistant asthma exacerbations. There were further increases in ER stress and UPR-related markers, airway neutrophilic inflammation, and various inflammatory mediators (i.e., type 2 cytokines, type 1/type 17 cytokines, epithelium-derived cytokines) in the lung of poly I:C-exacerbated OVALPS-OVA mice compared with those in OVALPS-OVA mice, all of which were closely associated with PKR phosphorylation. In fact, PKR has been known to stimulate various inflammatory pathways partly through NF-κB activation in the lung [59,60]. Therefore, it is possible that ER stress-related NF-κB activation may be closely associated with PKR in mediating CS resistance in the lung.

**Figure 2:**
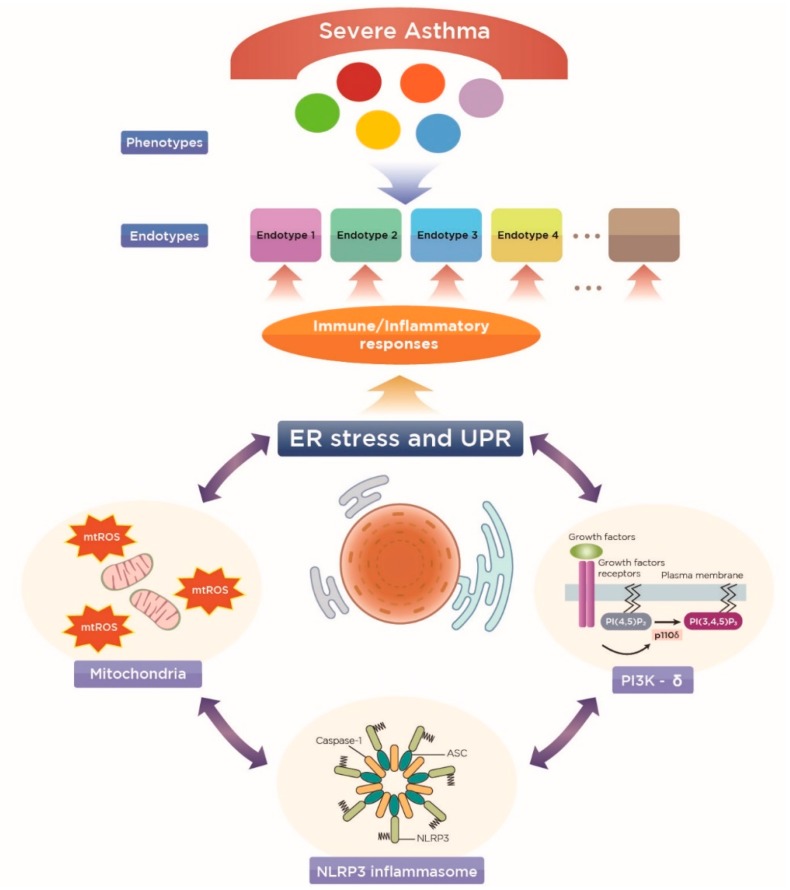
A novel concept of endotyping heterogeneous severe asthma based on the endoplasmic reticulum (ER) stress and unfolded protein response (UPR) and the ER stress-associated molecular pathways (mitochondria, NLRP inflammasome, and phosphoinositide 3-kinase (PI3K)-δ pathways), all of which are known to be closely implicated in corticosteroid-resistant inflammation in the lungs.

More importantly, ER stress-associated NF-κB can also be closely linked to cellular oxidative stress, a well-known inducer of CS refractoriness in the lung [61], mainly from mitochondria, another important subcellular organelle. Furthermore, in this process, there is close collaboration between these subcellular organelles and several cellular immune/inflammatory platforms known to induce CS resistance in the lungs (Figure 2), as described below.

## 5. Implications of Mitochondria and the Related Cellular Immune/Inflammatory Platforms in ER-Associated Endotypes of Severe Asthma

### 5.1. Mitochondria

Oxidative stress is one of the key features in chronic airway diseases including bronchial asthma [61]. Reactive oxygen species (ROS) can activate a broad range of cellular signaling, interact with biomolecules (e.g., lipids, proteins) producing secondary mediators, and cause protein modification and DNA damage, thereby inducing and maintaining the cardinal features of allergic airway inflammation. Importantly, oxidative stress is thought to crucially underlie molecular mechanisms leading to CS resistance in the lungs [62]. Mitochondria are regarded as one of the most powerful sources of intracellular ROS through the mitochondrial respiratory chain (mitochondrial ROS, mtROS) and they are tightly regulated in cells with regard to both generation and elimination [63]. However, when disturbed, subsequent development of oxidative stress can profoundly impact on the protein-folding capacity of the ER, as well as the whole cellular physiology, given that mitochondria function as signaling organelles [64]. Indeed, ER stress and mtROS are closely interconnected. An increase in Ca^2+^ leakage from the ER lumen in response to ER stress or oxidative stress leads to the accumulation of Ca^2+^ in the mitochondria, which results in decreased functional integrity of mitochondria and further generation of mtROS. Subsequent exacerbation of cellular oxidative stress causes more intensive Ca^2+^-release from the ER, perpetuating oxidative stress as a vicious cycle [65]. 

mtROS seem to play a key role in both severe type 2 and severe non-type 2 asthmatic features, particularly in mediating CS resistance in severe asthma. In a murine model of eosinophilic CS-resistant asthma (Af-exposed murine model with severe type 2 response profiles), significant increases in the production of mtROS are observed in the lung of Af-exposed mice (BAL cells) and Af-stimulated tracheal epithelial cells. Moreover, treatment with a potent mtROS scavenger, NecroX-5, significantly attenuates the Af-induced increases in ER stress and CS-resistant eosinophilic allergic lung inflammation [56]. A similar phenomenon is present in the OVALPS-OVA murine asthma model, wherein a mitochondrial-specific ROS scavenger remarkably ameliorates the CS-resistant neutrophilic non-type 2 immune response [66]. However, administration of N-acetylcysteine (NAC), a representative conventional antioxidant, does not show any beneficial effects on CS-resistant asthmatic features in either murine model (unpublished data), implying the critical involvement of the mtROS–ER stress interrelationship in mediating the CS resistance of severe asthma. This may represent an endotype of severe asthma related to subcellular organelles. Recently, abnormalities in mitochondrial metabolic pathways [67] and dynamics (e.g., fusion and fission) [68] are increasingly reported to be another crucial player in the pathogenesis of asthma, although their contribution to CS-resistant pulmonary inflammation is still unclear. 

### 5.2. NLRP3 Inflammasome

ER stress can lead to the release of diverse damage-associated molecular patterns (DAMPs) from mitochondria (e.g., mtROS, mitochondrial DNA, ATP, Ca^2+^). These mitochondrial DAMPs can be effectively detected by the cytoplasmic pattern-recognition receptor, NLRP3 inflammasome, leading to cleavage of the proinflammatory IL-1 family of cytokines, such as pro-IL-1β, and subsequent generation of an IL-1β-mediated potent inflammatory response [69]. Although contradictory findings have been reported on the involvement of NLRP3 inflammasome in the pathogenesis of bronchial asthma [70,71], increasing evidence indicates that NLRP3 inflammasome activation may be one of the pivotal players in CS-resistant asthmatic features in both type 2 and non-type 2 severe asthma. For instance, the features of a severe non-type 2 immune response of OVALPS-OVA mice have been reported to be controlled by mtROS-associated NLRP3 inflammasome activation, and that blockade of IL-1β significantly attenuates CS-resistant asthmatic features in this model [66]. Consistent with this finding, blockade of NLRP3 inflammasome effectively ameliorates neutrophilic inflammation in the mouse models of Chlamydia and Haemophilus respiratory infection-mediated, ovalbumin-induced CS-resistant allergic airway disease [72]. Similarly, in human asthmatics, there is a significantly increased gene expression of NLRP3, caspase-1, and IL-1β in sputum analysis from neutrophilic asthma patients [73], and neutrophilic airway inflammation, disease severity, and steroid resistance are correlated with NLRP3 and IL-1β expression [72]. Meanwhile, we have recently demonstrated that mtROS-mediated NLRP3 inflammasome activation in airway epithelium is also critically implicated in Af-induced CS-resistant eosinophilic asthmatic features [74], highlighting the potential of ER stress–mtROS-mediated NLRP3 inflammasome activation in airways as a unique endotype of severe asthma, irrespective of predominant airway inflammatory cell phenotypes. 

### 5.3. Phosphoinositide 3-Kinase-δ (PI3K-δ)

Phosphoinositide 3-Kinases (PI3Ks) are lipid signaling kinases that are frequently associated with cell membrane receptors such as growth factor receptors and cytokine receptors, and phosphorylate the 3’ position of inositol lipids to generate second messenger, phosphatidylinositol-3, 4, 5-trisphosphate (PIP3) at the plasma membrane [75]. Among the PI3Ks, the distribution of the delta isoform of class I PI3Ks is principally restricted to hematogenous inflammatory cells including circulating leukocytes and has been reported to play key roles in diverse immune/inflammatory processes including leukocyte signaling, antigen receptor signaling in T and B cells, mast cell degranulation, and migration and activation of neutrophils and eosinophils. In particular, PI3K-δ has been reported to be a key inducer of CS resistance in the lung, particularly associated with oxidative stress [56,62,74,76]. As for asthmatic features, we recently demonstrated that the blockade of PI3K-δ dramatically attenuated Af-induced CS-resistant type 2 allergic inflammation through the modulation of Af-induced ER stress and the related oxidative stress from mtROS, particularly in airway epithelium [56]. Further investigation on this therapeutic effect of PI3K-δ reveals that PI3K-δ modulates fungus-induced CS-resistant eosinophilic type 2 response through a close association with several critical CS-resistant inflammatory platforms including ER stress, mtROS, and NLRP3 inflammasome in airway epithelium [74]. However, our unpublished data show that the treatment effect of PI3K-δ blockade seems less clear in severe non-type 2 inflammation (e.g., OVALPS-OVA murine asthma model) compared with that seen in severe type 2 inflammation. On the basis of these findings, ER-associated PI3K-δ signaling may have potential as a novel endotype of CS-resistant severe type 2 immune response, rather than severe non-type 2 response. 

## 6. Lessons Learned from Sputum Transcriptomic Data of U-BIOPRED

The Unbiased Biomarkers for the Prediction of Respiratory Disease Outcomes (U-BIOPRED) consortium is a pan-European public–private collaboration, and attempts to stratify severe refractory asthma patients using an innovative system biology approach (e.g., “omics” including transcriptomic, proteomic, lipidomic, and metabolomic technologies), thereby providing a better template for personalized treatment of the disease based on pathobiological pathways [77,78]. Very recently, a clustering analysis of transcriptomic data from sputum cells obtained from 104 patients with moderate-to-severe asthma and 16 healthy volunteers has been reported [11]. In that study, they first defined a set of genes differentially expressed in the sputum of eosinophilic asthma (defined by asthmatic subjects with high sputum eosinophil counts ≥1.5%) and non-eosinophilic asthma (asthmatic subjects with low sputum eosinophil counts <1.5%) inflammatory phenotypes. Subsequent clustering of these genes revealed three transcriptome-associated clusters (TACs) distinguished by distinct sets of gene signatures (i.e. molecular phenotyping): TAC1, related to IL-13/TH2 and ILC2 signatures, blood and sputum eosinophilia, upregulation of receptors for TSLP, IL-33, IL-3, and CCL11 (CCR3); TAC2, associated with neutrophilic inflammation and having an inflammasome-dominant nature with IFN and TNF superfamily upregulation and higher expression of DAMPs; and TAC3, characterized by genes of metabolic pathways, ubiquitination, and mitochondrial functions, particularly for mitochondrial oxidative stress (OXPHOS) and the ageing process, pauci-granulocytic, and mild eosinophilia. These results are particularly important in that a clustering analysis using only clinical and physiologic parameters does not precisely differentiate patients with severe asthma, as mentioned above [7], and thus is not appropriate for developing the personalized treatment of severe asthma. In this context, data from U-BIOPRED transcriptomic analysis has incorporated a novel concept of molecular phenotyping involving different pathobiologic pathways with diverse clinical and physiological parameters. These results provide a brand-new framework for the development of specific treatments for severe asthma, particularly in relation to the ER and associated cellular immune/inflammatory platforms including mitochondria and inflammasomes. 

In addition, according to underlying inflammatory phenotypes, those TACs can be classified as eosinophil-predominant (TAC1 and TAC3), mixed granulocytic-dominant (TAC1 or TAC2), and neutrophil-dependent (TAC2 or TAC3) phenotypes. These inflammatory phenotypes together with gene expression patterns may shed light on the translation of experimental results on the pathogenesis of severe asthma.

TAC1 seems to involve mainly type 2 asthma including allergic (higher expression of IL-13/TH2 signatures) and non-allergic ILC2-mediated (higher expression of ILC2 signatures) eosinophilic asthma, which closely resembles the pathobiology of the fungi-induced (e.g., *Aspergillus* and *Alternaria* species) severe eosinophilic murine asthma model (i.e., related to IL-13/TH2 and ILC2 signatures, prominent tissue eosinophilia, upregulation of genes associated with epithelium-derived cytokine such as IL-33 and TSLP). Among non-type 2 phenotypes (TAC2 and TAC3), TAC2 may principally include non-type 2 severe asthma possessing a mixed type 1/type 17 neutrophilic immune response in the background of the variable extent of eosinophilic type 2 immunity [47]. In the U-BIOPRED clinical data, TAC2 was associated with a lesser extent of chronic airflow obstruction compared with that in TAC1; however, a mixed inflammatory nature is becoming increasingly known as a biomarker of the most severe form of asthma [10]. Experimentally, TAC2 resembles the inflammatory profiles of the OVALPS-OVA severe neutrophilic asthma model (i.e., mixed eosinophilic/neutrophilic inflammation; elevation of TH1 and TH17 cytokines including IFN-γ, TNF-α, and IL-17; activation of inflammasome) [54,66]. Lastly, TAC3 also represents another non-type 2 phenotype and it seems to have more complex regulatory factors than those of TAC1 and TAC2. Eosinophil-associated TAC3 may be more associated with inflammasome activation, which is quite different from the mechanism of TAC1 in mediating eosinophilic inflammation (through TH2 and ILC2 cells). In this context, the production of mitochondrial ROS in response to airway fungal exposure and related inflammasome activation in the fungal eosinophilic asthma model [74] may partly resemble the molecular phenotype of TAC3 experimentally. However, it is unclear how mitochondrial oxidative stress can be linked to asthma with little evidence of inflammation (pauci-granulocytic inflammation) and, at present, there does not seem to be an experimental system that can properly explain this phenomenon. Importantly, given that there is no approved endotype-driven therapeutic agent targeting the non-type 2 mechanism [13,14], these clustering results are quite valuable in suggesting possible pathobiologic mechanisms underlying non-type 2 severe asthma, namely TAC2 and TAC3, which are partly associated with inflammasome and mitochondria, respectively. Along with intensive experimental research on this issue, these clinical data may facilitate the future development of personalized treatments targeting non-type 2 severe asthma. 

Taken together, these clustering analysis data from severe asthma patients may provide us with a new framework for phenotyping the disease that incorporates underlying immune/inflammatory processes, particularly in association with ER-associated cellular inflammatory platforms (Figure 2), and for developing more effective and specific treatments, especially for non-type 2 severe asthma.

## 7. Conclusions

We now know that severe asthma is an extremely heterogeneous syndrome, rather than a single disease entity. In other words, the CS-resistant inflammatory nature of severe asthma may be driven by a variety of mechanisms wherein diverse cellular and molecular endotypes exist. These mechanisms have led to the current dichotomy of type 2 and non-type 2 pathways in the clinical and molecular aspects of severe asthma. Indeed, specific subgroups of severe asthma patients having eosinophil-predominant type 2 inflammation may derive benefit from the recent precision medicine targeting type 2 cytokines. However, there is no effective therapeutic modality, particularly for non-type 2 severe asthma, which comprises nearly 50% of all asthma patients. Notably, recent clinical data from a large European severe asthma cohort successfully incorporated molecular phenotyping involving different pathobiologic pathways, as well as diverse clinical and physiological parameters from severe asthmatic patients. The data presented a novel framework for proper disease endotyping and the development of specific treatments, particularly in relation to ER-associated cellular immune/inflammatory platforms including mitochondria and inflammasomes. In addition, the critical implications of these subcellular organelles in concert with several cellular immune/inflammatory platforms, such as NLRP3 inflammasome and the PI3K-δ pathway, in inducing CS resistance of the lungs are now being increasingly appreciated in numerous experimental models of severe asthma. These findings indicate that ER stress-associated molecular pathways may serve as a crucial endotype of severe asthma, and thus present a novel insight into the current knowledge and future development of treatments for heterogeneous severe asthma.
